# Physiopathological role of extracellular vesicles in alloimmunity and kidney transplantation and their use as biomarkers

**DOI:** 10.3389/fimmu.2023.1154650

**Published:** 2023-08-17

**Authors:** Elena Cuadrado-Payán, María José Ramírez-Bajo, Elisenda Bañón-Maneus, Jordi Rovira, Fritz Diekmann, Ignacio Revuelta, David Cucchiari

**Affiliations:** ^1^ Department of Nephrology and Kidney Transplantation, Hospital Clínic, Barcelona, Spain; ^2^ Laboratori Experimental de Nefrologia I Trasplantament (LENIT), Fundació de Recerca Clínic Barcelona-Institut d’Investigacions Biomèdiques August Pi I Sunyer (FRCB-IDIBAPS), Barcelona, Spain; ^3^ Red de Investigación Renal (REDINREN), Insituto de Salud Carlos III, Madrid, Spain

**Keywords:** extracellular vesicles, exosomes, kidney, transplant, kidney transplant, biomarker

## Abstract

Antibody-mediated rejection is the leading cause of kidney graft dysfunction. The process of diagnosing it requires the performance of an invasive biopsy and subsequent histological examination. Early and sensitive biomarkers of graft damage and alloimmunity are needed to identify graft injury and eventually limit the need for a kidney biopsy. Moreover, other scenarios such as delayed graft function or interstitial fibrosis and tubular atrophy face the same problem. In recent years, interest has grown around extracellular vesicles, specifically exosomes actively secreted by immune cells, which are intercellular communicators and have shown biological significance. This review presents their potential as biomarkers in kidney transplantation and alloimmunity.

## 1 Introduction

In kidney transplantation, antibody-mediated rejection (ABMR) continues to represent the most significant challenge to be resolved in order to improve graft and patient survival (1, 2). Although acute ABRM is a potentially treatable disease, chronic ABMR has limited therapeutic options. It invariably progresses to end-stage chronic kidney disease (ESKD), representing over 50% of death-censored graft losses. Therefore, early detection of acute ABMR, timely treatment, and prevention of its progression to chronic ABMR are vital to guarantee satisfactory results for kidney transplant recipients, especially in the high immunological risk group. With this premise, some centers have developed a strategy based on protocol kidney graft biopsies. However, a biopsy is an invasive method with potential risk of associated complications; moreover, it presents a high financial and resource cost (1, 2).

For this reason, many transplant centers choose to perform biopsies only “by indication” when some classical parameters are altered, such as creatinine, proteinuria, or the existence of specific anti-Human Leukocyte Antigen (HLA) donor antibodies (DSA) (3). However, these indicators need more sensitivity and change when rejection is established (3). This unmet clinical need led to a quest to discover early and sensitive biomarkers of graft damage, limiting renal graft biopsies’ performance to only those patients with a high likelihood of rejection. Furthermore, beyond the rejection field, other scenarios in kidney transplantation, such as delayed graft function (DGF) or interstitial fibrosis and tubular atrophy (IFTA), run into the same problem without biomarkers that allow for early detection or differentiation from other pathologies.

Among these possible biomarkers, it is worth highlighting circulating extracellular vesicles (EVs), specifically, exosomes actively secreted by immune cells, which are intercellular communicators that carry microRNA, DNA, and proteins with biological significance as intercellular mediators (4).

This review summarizes the current EVs literature in kidney transplantation and their use as biomarkers.

## 2 Types of EVs

The discovery of EVs dates to the last century. Since then, several names have been attributed to them, and a sharp increase in scientific publications has been evidenced in the last decade (5). In 2014, the Minimal Information for Studies of Extracellular Vesicles (“MISEV”) guidelines were released by the International Society for Extracellular Vesicles (ISEV). Subsequently, an update was proposed in 2018 by consensus of the largest group of EV experts, defining EVs as “the generic term for particles naturally released by cells that are delimited by a lipid bilayer and cannot replicate” (6). One year later, in 2019, another publication released by the corresponding authors of the MISEV guidelines asserted the accuracy and clarity of EV nomenclature to specialists and non-specialists and their use as a scaffold for progressively more detailed designation (7).

EVs are classified into three categories: exosomes, which are intraluminal vesicles contained in multivesicular bodies (MVBs) and released into the extracellular environment upon fusion of MVBs with the plasma membrane, microvesicles (also called microparticles) budded from the plasma membrane, and apoptotic bodies, the largest of the known vesicles and released during programmed cell death when the plasma membrane blebs (8–12) (**Table 1**).

**Table 1:** Characteristics of the different types of EVs (13–15).

Exosomes	Microvesicles	Apoptotic bodies
**Origin:** multivesicular bodies	**Origin:** plasma membrane	**Origin:** apoptotic cell death
**Size:** 50–100 nm	**Size:** 100–1,000 nm	**Size:** 1,000–5,000 nm
**Protein markers:** CD9, CD81, CD63, TSG101, ALIX	**Protein markers:** CD40 ligands, integrins, selectins, annexin V	**Proteins markers:** Histones

Due to the internal origin of exosomes from MVBs, they represent the parental cell’s internal activity and conditional state more closely than other types of EVs (5, 10, 11, 16, 17).

EVs seems that can be secreted by any cell type studied, including immune cells, are believed to play a central role in cell-to-cell communication (5, 6, 11). Their content is diverse and includes protein, lipids, nucleic acids, and other bioactive molecules that are determined according to the type of cell from which they arise (11, 16, 18). These content molecules provide EVs with different capabilities in terms of biogenesis and transport. Moreover, membrane curvature, which begins in the parent cell during membrane budding, determines the shape, composition, and size of each EV and therefore has a role in their physiological function (19–22).

Surface-exposed components and ligands determine EVs’ biodistribution and their binding to target cells or binding to the extracellular matrix, allowing intracellular signaling pathways via simple interaction with the surface of the target cells or by internalization. For instance, proteins such as tetraspanins (CD81, CD82, CD63, or CD9) help penetrate exosomes inside cells, invasion, and fusion, whereas heat shock proteins such as HSP70 and HSP90 are involved in antigen binding and presentation. Other proteins such as Alix, annexins, Rab, or TSG101 are associated with exosome release, membrane transport, and function. Notably, some of these proteins, such as CD63, CD81, HSP70, and TSG101, which are enriched explicitly in exosomes, are generally used as their marker proteins (13, 17, 18)

Lipids such as cholesterol and sphingomyelin enrich EV membranes and, as well as their essential structural role, can also be transferred between cells (23).

The parent cell source and the properties of target cells determine EVs’ biodistribution, and their quantity in circulation is determined by the balance between production and clearance. Clearance occurs via interactions with target cells through endocytosis, phagocytosis, pinocytosis, or membrane fusion (14), and also through the liver, spleen, and lungs (24, 25). Regarding the half-life, different studies have described a predominantly short one, ranging from 2–5 min to 5 h (24–27).

## 3 Isolation techniques

Up to now, there is no unique standardized protocol for EV isolation (28), and obtaining highly pure EVs is necessary to attribute them a specific function or property to be used as biomarkers (6). Their isolation and purification are decisive for most downstream applications due to their overlap with lipoproteins or protein aggregates; these can easily mistake EV detection due to their similar biophysical properties and act as contaminants (5, 15, 28).

EVs can be isolated from many sources, including biological fluids and cell culture supernatants (29, 30). Initial publications on blood-derived EVs prompted the recommendation to preferably conduct plasma studies due to platelet-derived EV released in the serum after blood collection during clot formation (30). In contrast, other studies have found EV isolation from serum to be more reproducible (29) and, in kidney transplantation, the serum content could reflect renal and endothelial recovery functions (31, 32)

According to MISEV 2018, differential ultracentrifugation was the most common isolation technique (6). It consists of consecutive centrifugation steps, each with an increase in centrifugation forces and durations, aiming to isolate smaller from larger particles based on differences in their densities (33). Other procedures, such as size exclusion chromatography (SEC), ultrafiltration, precipitation, or immunoisolation, were used by approximately 5–20% of researchers (6).

Since then, an assortment of techniques has been developed, such as field-flow fractionation (FFF), variations of size exclusion chromatography (SEC), ion exchange chromatography, microfiltration, fluorescence-activated sorting, novel immunoisolation techniques, and microfluidics or precipitation techniques using a plethora of commercial kits (5, 6).

Further information on EV isolation is beyond the scope of this review. All these methods, along with their strengths and weaknesses, are extensively discussed elsewhere (34, 35).

## 4 Biological function and role in immunology

EVs are involved in the regulation of physiological functions such as maintenance of homeostasis, enhanced coagulation (36–38), vessel integrity (39), tissue repair (40), or synaptic plasticity (41). They are also involved in inflammation, angiogenesis, or transmission of oncogenic molecules to neighboring cells, favoring neoplasia propagation and procoagulant capacity (4, 10, 11, 42–44).

Regarding their role in immunology, EVs act in innate and adaptative immune systems. In the innate, their major role has been described as pro-inflammatory mediators secreted by activated macrophages, neutrophilic granulocytes, NK cells, or mast cells in scenarios such as infection (45–47), sepsis (47–49), or chronic inflammation (50). In addition, an anti-inflammatory role also has been described through TGF-β secretion or regulation of endogenous lipid mediators (51).

Regarding adaptive immunity, EVs are a source of antigens for antigen-presenting cells (APC) such as macrophages, dendritic cells (DCs), and B cells. Depending on their cargo and parenting cells, they can induce immunogenic or tolerogenic responses (8, 42, 52). Recipient APCs can release EVs containing peptide-MHC I or II complexes and co-stimulatory molecules that contribute to antigen presentation (53, 54). This release is carried out constitutively, although this process can be increased after stimulation (55). Furthermore, graft-derived exosomes can initiate the immune response in a direct or semi-direct pathway that will end up causing graft rejection (56, 57). The direct pathway consists of exosomes from donor tissue behaving as donor APCs presenting MCH molecules or tissue-specific self-antigens to recipient T cells (58, 59). In contrast, in the semi-direct pathway, graft exosomes are taken up by recipient APCs, presenting intact MHC molecules from these graft exosomes on the surface of APCs, known as MHC cross-dressing (60).

Besides promoting intercellular information exchange via their surface molecules, their role as carriers of soluble mediators such as cytokines has been described. These cytokines include interleukin 1β (IL-1 β), interleukin 1α (IL-1 α), interleukin 18 (IL-18), macrophage migration inhibitory factor (MIF), interleukin 32, membrane-bound tumor necrosis factor (TNF), interleukin 6 (IL-6), vascular endothelial growth factor (VEGF), interleukin 8 (CXCL8), fractalkine (CX3CL1), CCL2, CCL3, CCL4, CCL5, and CCL20, and transforming growth factor β (TGF β) (27, 61).

## 5 Extracellular vesicles as biomarkers in kidney transplantation

Nowadays, biomarkers of the different EVs in circulation have been assessed in plenty of publications. The most developed field is oncology, where tumor mass has been linked to the amount of tumor-derived circulating exosomes. In the field of kidney disease, some studies demonstrate the participation of exosomes in different processes, which include acute kidney failure, autoimmune kidney disease, diabetic kidney disease, glomerulonephritis, vasculitis, or thrombotic microangiopathies (16).

Regarding transplantation, EVs in body fluids have been proposed as liquid biopsies. Mainly, publications focus on heart, lung, or pancreatic islet allografts. A profile of blood-derived EVs through multiplex flow cytometric assay using antibody-coated capture beads has been described in heart transplant recipients. A significant increase in the concentration of plasma-derived EVs in patients undergoing both acute cell and ABMR has been confirmed compared with subjects not undergoing them (62). In the lung, circulating exosomes with lung self-antigens can be a viable non-invasive biomarker for identifying patients at risk of developing chronic lung allograft dysfunction (63–65). Regarding pancreatic islet allografts, a human-into-mouse xenogeneic islet transplant model led to a marked decrease in the transplant islet exosome signal in early rejection, and changes in exosomal microRNA and proteomic profiles preceded hyperglycemia (66).

**Figure 1:**
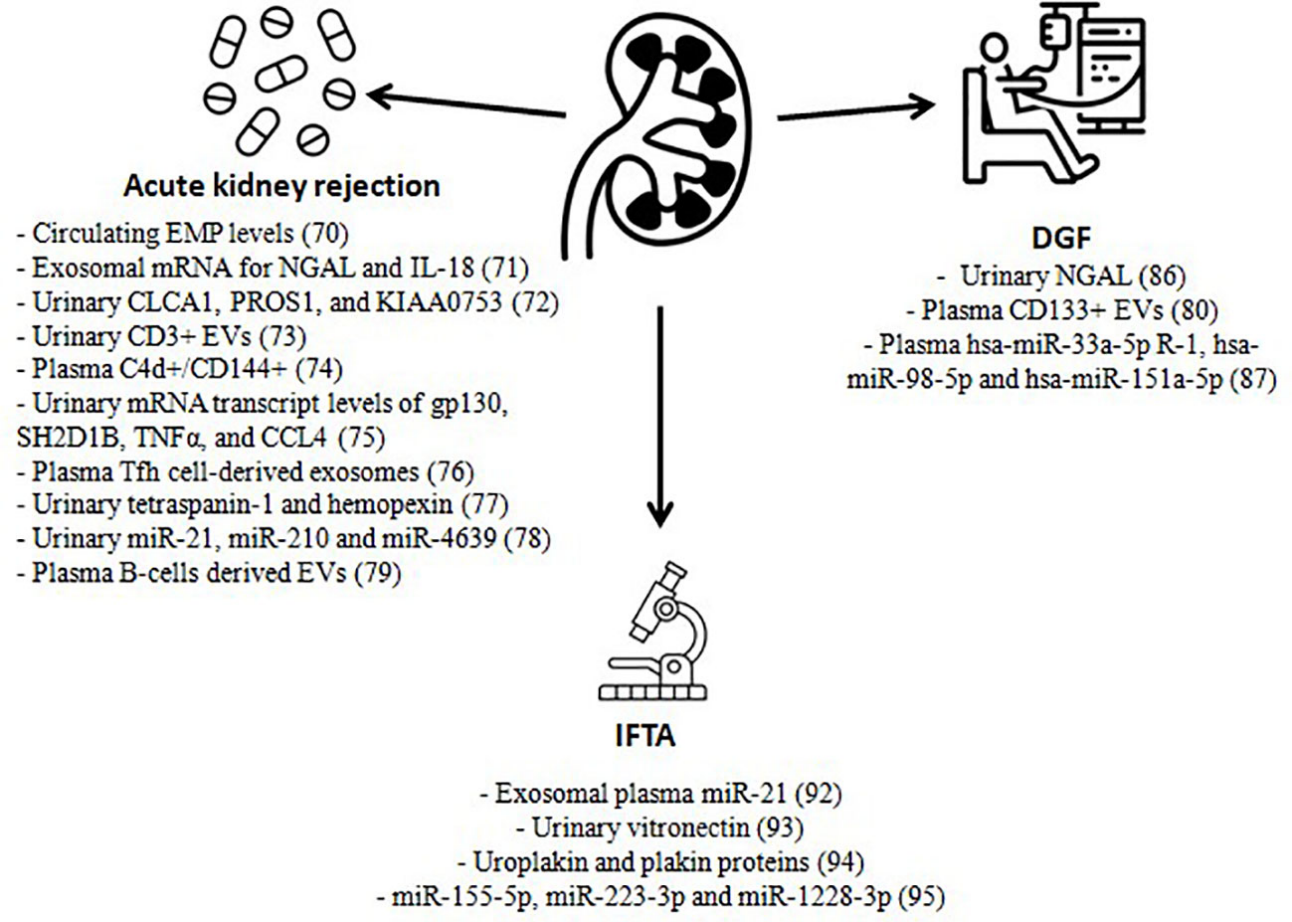
Extracellular vesicles as biomarkers in kidney transplantation. EMP, endothelial microparticles; NGAL, neutrophil gelatinase-associated lipocalin; IL-18, interleukin 18; EVs, extracellular vesicles; Tfh, T follicular helper; gp130, glycoprotein 130; SH2D1B SH2, domain containing 1B, TNFα, tumor necrosis factor alpha; CCL4, chemokine ligands 4; DGF, delayed graft function; IFTA, interstitial fibrosis and tubular atrophy.

In kidney transplant, a decrease of circulating microparticle levels and their procoagulant activity after graft has been described in comparison to the prior hemodialysis status, hypothesizing that microparticles could be associated with cardiovascular risk improvement after transplant (67, 68). Studies have also been carried out on the urine and plasma of recipients, revealing their potential use as markers of cellular or humoral rejection, DGF, IFTA, mediated drug toxicity, and other non-specific graft injuries (18). Below, we expand the role of EVs in all of these settings (**Figure 1**). The methods of isolation for each study are summarized in **Table 2**.

**Table 2:** Methods of EV isolation in each study mentioned.

Study	EV isolation method
Qamri et al. (69)	Centrifugation
Peake et al. (70)	Centrifugation
Sigdel et al. (71)	Ultrafiltration
Park et al. (72)	Differential ultracentrifugation + immunoisolation
Tower et al. (73)	Centrifugation
Zhang et al. (74)	Precipitation - exoRNeasy serum/PlasmaMidi Kit
Yang et al. (75)	Precipitation - ExoQuickTM Kit (SBI Corporation)
Lim et al. (76)	Ultracentrifugation
Chen et al. (77)	Size-exclusion chromatography
Cucchiari et al. (78)	Size-exclusion chromatography
Alvarez et al. (79)	Ultracentrifugation
Dimuccio et al. (80)	Ultracentrifugation
Wang et al. (81)	Precipitation - exoEasy Maxi Kit (Qiagen)
Saejong et al. (82)	Precipitation - polyethylene glycol (PEG)
Carreras-Planella et al. (83)	Size-exclusion chromatography
Carreras-Planella et al. (84)	Size-exclusion chromatography
Costa de Freitas et al. (85)	Precipitation - miRCURY Exosome Kit (Qiagen)

### 5.1 Biomarkers for acute kidney rejection

In graft rejection, all the immune system components are involved in causing graft injury, including T and B lymphocytes, antibodies, endothelial cells, NK cells, macrophages, polymorphonuclear cells, or complement components (86).

Qamri et al. analyzed early post-transplant changes in circulating endothelial CD31+/CD42b− microparticle (EMP) levels after kidney transplant in 213 kidney recipients and 14 kidney + pancreas recipients. In the first cohort, no changes in circulating EMP levels were observed when graft dysfunction was unrelated to acute rejection. However, when this dysfunction was due to an episode of acute rejection (confirmed through a graft biopsy), an elevation in circulating EMP was detected. At the time of stratification according to PTC C4d staining, in patients with the negative one, a faster decrease in EMP levels was observed in comparison with patients with positive PTC C4d staining. This led the authors to suggest that circulating EMP levels could inform about ongoing endothelial cell injury. Moreover, when analyzing the different etiologies of end-stage kidney disease, a trend was found toward a decline in post-transplant EMP levels in causes due to diabetes mellitus, obstructive/inherited kidney disease, and autoimmune disease (69).

In another study by Peake et al., urinary exosomal mRNA from frequent kidney injury biomarkers such as neutrophil gelatinase-associated lipocalin (NGAL), interleukin-18 (IL-18) and kidney injury molecule-1 (KIM-1), together with the constitutively produced cystatin C, were compared with their corresponding synthesized urinary protein levels as well as with the creatinine reduction ratio (CRR) on post-operatory day 7. The results showed that, although urinary NGAL and IL-18 protein levels did correlate with CRR on day 7, this was not the case for mRNA inside urinary exosomes. The explanation for these findings lay in the selectivity for exosome packaging and does not have to be representative of the parenting cell (70).

One year later, Sigdel et al. described 11 proteins to be enriched in the urinary exosomes of patients with biopsy-proven acute rejection; three of these proteins (CLCA1, PROS1, and KIAA0753) had not been previously identified in healthy urine exosomal proteins (71).

Park et al. reported a urine-based platform, iKEA (integrated kidney exosome analysis), to detect rejection of kidney transplants through T-cell-derived EVs. This platform, based on a magneto-electrochemical sensing system, revealed a higher level of CD3-positive EVs in kidney rejection recipients, with an accuracy of approximately 91.1% (72).

Tower et al. found a correlation between plasma C4d+, especially C4d+/CD144+ microvesicles, and the presence of ABMR and its severity and response to treatment in kidney recipients. Ninety-five kidney recipients with for-cause biopsies performed and twenty-three healthy volunteers were evaluated. After histopathologic examination of the graft biopsies, 28 patients with ABMR were found. In them, the density of C4d+/CD144+ microvesicles was 11-fold greater than in kidney transplant patients without ABMR and 24-fold greater than in healthy volunteers. The densities of C4d+ and C4d+/annexin V+ (C4d+/AVB+) microvesicles were also higher in ABMR recipients. Moreover, C4d+/AVB+ microvesicles correlated with ABMR biopsy severity. Lastly, in nine cases, treatment was associated with a reduction in the densities of C4d+/CD144+ and CD144+ microvesicles, which also showed them to be a treatment response monitoring tool (73).

Zhang et al. selected 21 genes (related to inflammatory and IL-6 signaling events or elevated in renal biopsies of patients with ABMR) whose mRNA transcript levels were increased in plasma exosomes of ABMR kidney recipients compared with cell-mediated rejection and/or no rejection. The authors also generated a gene score with the combination of the transcript levels of four of these genes (gp130, SH2D1B, TNFα, and CCL4), which was significantly higher in the ABMR group than the other groups (74).

Yang et al. suggested a correlation between ABMR and follicular helper T cell (Tfh cell)-derived exosomes through their increase in the circulation of such patients compared with non-ABMR patients. Moreover, Tfh cell-derived exosomes promoted B cell proliferation and differentiation. Moreover, their study reported a decline in CTLA-4 expression on the Tfh cell-derived exosome surface in kidney transplant patients with ABMR. CTLA-4 is a leukocyte differentiation antigen and a transmembrane receptor on T cells, with an established role in alloantigen-driven T cell activation and various autoimmune diseases. CTLA-4 on exosomes inhibited human T cell activation by directly interacting with the molecules CD80 or CD86. Furthermore, intracellular CTLA-4 can inhibit Tfh cell differentiation, reduce IL-21 secretion, and inhibit B cell proliferation and differentiation into plasma cells (75).

Regarding acute T cell-mediated rejection, Lim et al. identified several urinary exosomal biomarker candidates in an Asian population of kidney transplant patients using a proteomics approach. Validation of the findings by western blot assay proved that tetraspanin-1 and hemopexin were significantly higher in TCMR patients (76).

Chen et al. established a circulating exosomal miRNA panel by extracting plasma exosomes from 58 kidney transplant recipients and 27 healthy controls. Exosomal miR-21, miR-210, and miR-4639 could discriminate between subjects with chronic kidney dysfunction and those with normal graft function. At one year follow-up, patients with a low calculated score from this three-miRNA panel revealed a stable recovery of allograft function (77).

Lastly, our group has proposed using B-cells–derived EVs (BEVs) to check B cell proliferation in secondary lymphoid organs and bone marrow after desensitization. BEVs (CD19+ or HLA-II+) were associated with surviving B cells in lymph nodes retrieved upon surgery on patients who received desensitization with Rituximab, plasma exchanges, and immunoglobulins. After the administration of Rituximab, no B cells were circulating. CD19+ or HLA-II+ EVs may reflect the mass of surviving B cells in secondary lymphoid organs that may predispose them to subsequent rejection. This is suggested by the rebound of BEVs in patients who develop ABMR after desensitization (78).

### 5.2 Biomarkers for DGF

DGF leads to a higher risk of acute rejection and progression to chronic graft dysfunction (80, 87–89). The leading cause of DGF, ischemia-reperfusion injury (IRI), prompts a complex, alloantigen-independent immune response, which includes crosstalk between polymorphonuclear cells, macrophages, and donor cells as well as the release of EVs with pro-inflammatory and anti-inflammatory effects (80, 90, 91). Moreover, endothelial cells and renal tubular epithelial cells release EVs when exposed to oxidative stress, hypoxia, an acidic environment, or inflammation (8, 90, 91).

Among the first studies on exosomes and kidney dysfunction, Alvarez et al. evaluated if the different urine fractions (cellular or exosomal) have different NGAL expression in 15 kidney allograft recipients (eleven living donors and four deceased donors) soon after transplantation. Western blot analysis showed that the average NGAL expression in the exosomal fraction was significantly higher in deceased donor patients from the first post-operatory day and that its expression lasted increased in patients with DGF compared with non-DGF patients (79).

Dimuccio et al. showed lower levels of CD133-positive EVs in urine samples of transplanted patients. This decrease was evidenced from the first post-operatory day until day 7, when an increase was described. However, compared with patients with DGF, these last had a significant rise. Moreover, in patients with severe pre-transplant vascular injury of the allograft, CD133-positive EVs did not increase. The origin of CD133-positive EVs appeared restricted to glomeruli and proximal tubules. These data implicate CD133-positive cells in renal tissue regeneration after injury due to cold ischemia and IRI. Accordingly, no increase was observed in recipients with severe pre-transplant vascular damage, implying an inefficient regeneration of the graft tissue in these recipients (80).

In another study, Wang et al. explored miRNA expression profiling in the DGF process. Fifty-two known and five conserved exosomal miRNAs expressed in kidney-transplanted patients with DGF were identified. Three co-expressed exosomal miRNAs: hsa-miR-33a-5p R-1, hsa-miR-98-5p, and hsa-miR-151a-5p, were further observed to be significantly upregulated in the peripheral blood of DGF patients. Moreover, hsa-miR-151a-5p was positively correlated with the patient’s first-week serum creatinine levels, blood urea nitrogen, and uric acid after transplantation (81).

### 5.3 Biomarkers for interstitial fibrosis and tubular atrophy

In the field of kidney transplant, fibrosis serves as the final and irreversible stage of the pathogenic mechanisms that lead to the loss of allograft function (92). For this reason, beyond the invasiveness of renal biopsy, clinical data need to be more specific to allow for early detection (92–95).

Saejong et al. describe the potential use of microRNA (miR)-21 expression in plasma exosomes for non-invasive monitoring of high-grade IFTA in kidney transplant patients. There are already previous studies on the role of exosomal miR-21 as a fibrosis biomarker and its association with TGF-β, a cytokine known to be involved in fibrosis pathogenesis. In the study by Saejong, miR-21 from the plasma exosome fraction (but not from the whole plasma) could discriminate between low- versus high-grade IFTA. It is demonstrated that the released miR-21 decreases phosphatase and tensin homolog (PTEN), which causes the phosphorylation of Protein kinase B (AKT) signaling, in turn reducing the expression of E-cadherin and raising the expression of α-SMA and fibronectin in kidney tubules (82).

More recently, Carreras-Planella et al. describe the search for kidney allograft dysfunction protein biomarkers related to four biopsy-proven diagnoses: IFTA, acute T-cell rejection, calcineurin inhibitors toxicity, and normal kidney function. The authors carried out a proteomic analysis of the urinary EVs (uEVs), discovering some EV-associated proteins that show different expressions depending on whether they come from pathological or normal kidney function allografts. Moreover, a change in the expression of vitronectin (VTN) was also evidenced in recipients with chronic IFTA, suggesting urinary VTN levels as another possible biomarker for monitoring fibrotic changes in kidney transplant patients. For the fibrosis process to occur, VTN must join the potent profibrotic glycoprotein PAI-1, although the precise mechanisms are arguable. VTN has been reported to increase PAI-1 activity in the renal tissue, hindering fibrinolysis. However, other studies described the opposite, highlighting a protective role against fibrosis (83).

The same group also demonstrated the potential role of uEVs as biomarkers of chronic calcineurin inhibitor toxicity (CNIT). Their nephrotoxicity and role in kidney fibrosis are known and have been described in multiple studies and they are first-line agents in the immunosuppressive regimen of kidney transplantation. The problems we continue to face are CNIT diagnosis and management. In this study, the urine from kidney transplant recipients with CNIT diagnosis is compared with recipients with IFTA and without CNIT or normal allograft function (all of them under a similar immunosuppressive scheme that included CNI). After data analysis, members of the uroplakin (UPK1A, UPK1B, UPK2, and UPK3A) and plakin families (periplakin and envoplakin) were significantly upregulated in the CNIT group, suggesting a central role in CNIT development. The binding of uroplakin proteins covers the urothelium’s surface to prevent urine influx from the lumen, also covering the renal pelvis, ureters, urinary bladder, and prostatic urethra. Periplakin and envoplakin function as cell-linker proteins. The upregulation of these proteins in the CNIT recipient’s uEV suggests that the toxic effect of CNI on the urothelium may increase their citolinker activity (84).

Lastly, Costa de Freitas et al. also evaluated the expression of different urinary exosome-derived miRNAs (exo-miRs) in transplant patients on a tacrolimus regimen. As a result, a difference in the expression of 16 exo-miRs was observed. Among them, the marked upregulation of miR-155-5p and downregulation of miR-223-3p and miR-1228-3p stand out. Moreover, it was found that the tacrolimus dose correlated with the expression of miR-155-5p and miR-223-3p, serum creatinine with the expression of miR-223-3p, and the number of blood leukocytes with miR-223-3p and miR-1228-3p (85).

## 6 Discussion

EVs participate in intercellular communication in physiological and pathological processes, and in recent years, interest in them has grown as tools to monitor post-transplant evolution in a non-invasive way. Previous studies on diverse biological samples (blood or urine) include a wide range of pathologies such as kidney graft rejection (both cellular and humoral), DGF, IFTA, and drug toxicity. The limitations we have to consider are that the studies published and presented here do not often include multiple centers, the number of patients included is low, and the results have yet to be validated in larger cohorts. Appropriate method validation studies need to be improved, and the isolation protocol needs standardization to avoid the co-isolation of various vesicles or differences in contamination levels. The most modern technologies will likely offer new opportunities in this field; for instance, the Imaging Flow Cytometry (IFCM)-based methodology for the direct detection (without prior isolation) of donor-derived EVs (dd-EVs) in the plasma of kidney transplant patients based on Human Leukocyte Antigen (HLA) mismatch (96) or further investigation into the proteomic landscape and protein signature in urinary EVs (97).

Despite the promising published data, nowadays, we cannot use EVs as a definitive decision tool. Future studies are required before their analysis could facilitate the decision process in routine clinical practice. We still need basic parameters such as creatinine, proteinuria, or specific anti-HLA donor antibodies, and EV analysis may not replace the invasiveness of graft biopsy as the gold standard.

Future studies will extend our knowledge of the role of EVs as biomarkers in the kidney transplant field. A combination of biomarkers could help us decide whether a biopsy should be done and may have a supportive role when interpreting data provided by an allograft biopsy.

## Author contributions

Conceptualization, EC-P, MR-B, EB-M, JR, and DC; resources, EC-P, MR-B, EB-M, JR, and FD; writing—original draft preparation, EC-P and DC; writing—review and editing, IR and DC; supervision, IR. All authors contributed to the article and approved the submitted version.
